# Safety and efficacy of a modified busulfan/cyclophosphamide conditioning regimen incorporating cladribine for autologous hematopoietic stem cell transplantation in acute myeloid leukemia

**DOI:** 10.3389/fphar.2023.1014306

**Published:** 2023-02-03

**Authors:** Yuan-Yuan Shi, Zeng-Yan Liu, Gui-Xin Zhang, Yi He, Ming-Zhe Han, Si-Zhou Feng, Rong-Li Zhang, Er-Lie Jiang

**Affiliations:** ^1^ State Key Laboratory of Experimental Hematology, National Clinical Research Center for Blood Diseases, Haihe Laboratory of Cell Ecosystem, Institute of Hematology & Blood Diseases Hospital, Chinese Academy of Medical Sciences & Peking Union Medical College, Tianjin, China; ^2^ Department of Hematology, Binzhou Medical University Hospital, Binzhou, China

**Keywords:** Cladribine, Acute Myeloid Leukemia, Autologous hematopoietic stem cell transplantation, Conditioning regimen, efficacy and safety

## Abstract

This is a small phase I study examining the safety and efficacy of a cladribine (CLAD)-containing conditioning regimen prior to autologous hematopoietic stem cell transplantion (auto-HSCT) for patients with acute myeloid leukemia (AML). All patients, aged 15–54 years (median 32 years), had favorable/intermediate risk AML (n = 20) or acute promyelocytic leukemia (APL; n = 2) and no evidence of minimal residual disease (MRD) prior to transplantation. Fourteen of the 22 patients received the conditioning regimen as follows: busulfan (Bu) + cyclophosphamide (Cy) + CLAD + cytarabine (Ara-c) or idarubicin. The conditioning regimen of 8 patients was without Cy nor idarubicin to reducing adverse cardiac reaction: the regimen of Bu + CLAD+ Ara-c for 6 patients; and the regimen of Bu + melphalan + CLAD + Ara-c for the other 2 patients. All 22 AML patients received peripheral blood stem cell transplantation. The number of infused mononuclear cells and CD34^+^ cells was 10.00 (2.88–20.97) × 10^8^/kg and 1.89 (1.52–10.44) × 10^6^/kg, respectively. Hematopoietic reconstitution was achieved in all patients, with a median time of 13 (10–34) days for neutrophils and 28 (14–113) days for platelets. Two patients suffered from pulmonary infection, 4 patients suffered from septicemia during the neutropenic stage, and the others suffered from infection or gastrointestinal reaction without exceeding grade 3 after conditioning, which were all alleviated by anti-infection and other supportive treatment. None of the patients died of transplantation-related complications. At a median follow-up of 29.5 (ranging from 4.0 to 60.0) months, three patients relapsed after auto-HSCT at a median time of 6 (ranging from 0.5 to 10.0) months. One patient died due to relapse at 18 months after auto-HSCT. The remaining 21 patients were all alive, including 19 patients with negative MRD. The other 2 patients achieved negative MRD after allogeneic HSCT or chemotherapy. The estimated 2-year survival, relapse, and Leukemia-free survival rates were 94.1 ± 5.7%, 14.7 ± 7.9% and 85.3 ± 7.9%, respectively. A CLAD-combination conditioning regimen is efficient and safe for auto-HSCT, indicating an effective approach for AML treatment.

## Introduction

Consolidation regimens for patients with acute myeloid leukemia (AML) after complete remission (CR) include conventional chemotherapy, autologous hematopoietic stem cell transplantation (auto-HSCT), and allogeneic hematopoietic stem cell transplantation (allo-HSCT). Allo-HSCT is the preferred treatment for AML in adults with a poor prognosis. In contrast, the choice of consolidation therapy for AML with a favorable prognosis and intermediate prognosis is still controversial. Auto- HSCT has fewer transplant complications, lower transplant-related mortality, and higher quality of life after transplantation. Compared with conventional chemotherapy, auto-HSCT can reduce the recurrence rate and improve the overall survival (OS) rate of AML patients (Cornelissen et al., 2015; Heini et al., 2017). Therefore, auto-HSCT therapy can be used for AML patients lacking allogeneic donors and obtaining CR. Compared with allo-HSCT, auto-HSCT has the disadvantage of high recurrence risk. Reducing the recurrence rate after auto-HSCT is one of the keys to further improving the efficacy of auto-HSCT. The main reason for recurrence after auto-HSCT is that the remaining leukemia cells in patients are not entirely cleared. Therefore, optimizing the conditioning regimen may be an effective means to reduce relapse after auto-HSCT. Cladribine (CLAD) is a purine nucleoside analog that can kill both proliferating and nonproliferating cells and promote apoptosis (Vahdat et al., 1994; Robak and Wierzbowska, 2014; Freyer et al., 2015). When combined with cytarabine (Ara-c), it can enhance the cytotoxicity of Ara-c to achieve antitumor effects (Ramos et al., 2015). In patients with relapsed and refractory AML, CLAD showed a good anti-leukemia effect. Compared with traditional chemotherapy, CLAD-combination chemotherapy can significantly improve patients' remission rate and survival rate (Holowiecki et al., 2012). Therefore, we adopted a CLAD-combination conditioning regimen in auto-HSCT to enhance the antileukemia effect. Auto-HSCT has been performed in 22 AML patients and achieved good efficacy, which is reported as follows.

## Patients and methods

### Patient characteristics

Definition: (1) Complete remission (CR): bone marrow morphological analysis showed that leukemia cells were <5%, hemogram normalized, and there was no extramedullary leukemia (Shen and Zhao, 2018). (2) minimal residual disease (MRD): multicolor flow cytometry was used for analysis, with a detection sensitivity of 10^−4^; negative was defined as no leukemia cells detected by flow cytometry (<0.01%); fluorescence quantitative polymerase chain reaction (FQ-PCR) was used to analyze AML1-ETO, CBFβ-MYH11, and other fusion genes, and the sensitivity of detection was 10^−5^∼10^−6^; negative was defined as no residual leukemia detected by FQ-PCR (<0.0032%). (3) Relapse of leukemia after transplantation: Relapse was defined as one of the following: MRD positive, morphologic relapse (bone marrow smear, with morphology showing leukemic cells >5%), or extramedullary relapse.

Inclusion criteria: (1) nonpoor risk stratification of adult AML patients who received auto-HSCT in the transplant center of our hospital from September 2016 to May 2021; (2) age 15–54 years; (3) disease state before transplantation at the CR stage; (4) negative MRD of bone marrow and samples before transplantation; and (5) CLAD-combination conditioning regimen.

### Diagnosis and risk stratification

All patients were diagnosed according to the results of cytomorphology, cytogenetics, molecular biology, and immunophenotype, and the diagnostic classification criteria were based on the FAB and WHO (2008 edition) standards (Vardiman et al., 2009; Shen and Zhao, 2018). Risk stratification for AML according to the ELN 2017 classification (Dohner et al., 2017).

### Treatment

Induction and consolidation therapy: 1) Induction and consolidation chemotherapy of AML for non acute promyelocytic leukemia (APL): anthracycline [daunorubicin (DNR) 45 mg/m^2^/d] was used in newly diagnosed patients, d1-3, or idarubicin (IDA) 10 mg/m^2^/d, d1-3] and Ara-C (100–200 mg/m^2^/d, d1-7) regimen-induced therapy. Consolidation chemotherapy was performed after CR acquisition, and the regimen included medium dose/high dose Ara-C (2–4 g/m^2^/d, d1-3) ± anthracycline [DNR 45 mg/m^2^/d, d1-3, or IDA 10 mg/m^2^/d, d1-3, or homoharringtonine (HHT) 2.5 mg/m^2^/d, d1-7, or mitoxantrone (MTZ) 8 mg/m^2^/d, d1-3], CAG [acramomycin 7 mg/m^2^/d, d1-8; Ara-C 10 mg/m^2^/d, d1-14; granulocyte colony stimulating factor (G-CSF) 200 ug/m^2^/d, d1-14], etc. After induction of remission, all patients were given triple [methotrexate (MTX) 10 mg + Ara-C 50 mg + dexamethasone (DEX) 10 mg] scabbed injection to prevent or treat CNS leukemia, with a median of 4 (2–5) scabbed injections before transplantation.

2) APL induction and consolidation chemotherapy: two patients with newly diagnosed APL were given all-trans retinoic acid (ATRA, 30 mg/m^2^/d) + arsenic trioxide (ATO, 10 mg/d, d1-28) + Ara-C (100 mg/m^2^/d, d1-7) or IDA (8 mg/m^2^/d×4d) for induction therapy, followed by ATO (10 mg/d×21d or 28 d) + ATRA (30 mg/m^2^/d) + anthracycline (DNR 45 mg/m^2^/d, d1-3 or IDA 8 mg/m^2^/d, d1-4 or MTZ 8 mg/m^2^/d, d1-3) for consolidation therapy and maintenance therapy with Fufang Huangdai tablets and ATRA (30 mg/m^2^/d). Combined chemotherapy was given after relapse, including ATO (10 mg/d×28 d) + IDA (8 mg/m2/d×4 d) ± ATRA (30 mg/m^2^/d) and multiple courses containing anthracyclines (DNR, 45 mg/m^2^/d, d1-3 or IDA, 10 mg/m^2^/d, d1-3 or HHT, 2.5 mg/m^2^/d, d1-7 or MTZ, 8 mg/m^2^/d, d1-3) combined with standard dose/medium dose Ara-c (100 mg/m^2^/d, d1-7 or 2 g/m^2^/d, d1-3).

Hematopoietic stem cell mobilization and collection: Patients received a medium-dose/high-dose Ara-C (2–4 g/m^2^/d, d1-3) ± anthracycline (DNR, 45 mg/m^2^/d, d1-3, or IDA, 10 mg/m^2^/d, d1-3) regimen for autologous stem cell mobilization. One week after chemotherapy cessation, G-CSF at 5–10 ug/kg/d was administered subcutaneously, and peripheral blood stem cells were collected in patients when leukocytes >5×10^9^/L. Peripheral blood stem cells were collected from 22 patients. All samples were evaluated by MRD, and auto-HSCT was performed only when MRD was negative.

Conditioning regimen and number of stem cells returned: All patients received myeloablative conditioning. The conditioning regimen of 14 AML patients was as follows: intravenous(i.v.) busulfan (Bu) 3.2 mg/kg/day, days −9,-8,-7, i.v. CLAD 10 mg/day, days −6,-5,-4, i.v. cyclophosphamide (Cy) 40 mg/kg/day, days −3,-2 and i.v. Ara-c 2 g/m^2^/day or IDA 12 mg/m^2^/day, days −6,-5,-4; the conditioning regimen of 6 AML patients was as follows: i.v. Bu 3.2 mg/kg/day, days −9,-8,-7, i.v. CLAD 10 mg/day, days −6,-5,-4,-3,-2, and i.v. Ara-c 2 g/m^2^/day, days −6,-5,-4,-3,-2; the conditioning regimen of another 2 AML patients was as follows: i.v. Bu 3.2 mg/kg/day, days −9,-8,-7, i.v. melphalan 70 mg/m^2^/day, days −3,-2, i.v. CLAD 10 mg/day, days −6,-5,-4 and i.v. Ara-c 2 g/m^2^/day, days −6,-5,-4. After conditioning, no less than 1.5×10^6^/kg autologous stem cells was transfused back into each patient at day 0.

Hematopoietic reconstitution and maintenance therapy: Definition of hematopoietic reconstitution: Myeloid reconstitution was defined as a neutrophil count ≥0.5×10^9^/L for 3 consecutive days, and Day 1 was the time of reconstitution; platelet reconstitution was defined as a platelet count ≥20×10^9^/L for 7 consecutive days without platelet infusion, and Day 1 was the time of reconstitution.

Maintenance therapy: after transplantation, leukocytes >3.0×10^9^/L and platelets >50×10^9^/L, some patients began to receive maintenance therapy for approximately 1 year. Among them, maintenance therapy with interleukin-2 (100 wu/d, QOD) or interferon (300 wu/d, QOD) and azacytidine (100 mg/d×5, 4-week course) was performed for 5 patients with intermediate prognosis and 2 patients with favorable prognosis, and sorafenib (0.2 g, 2 times/d) was applied for 1 AML with FLT3/ITD-positive patients with intermediate prognosis.

### Efficacy evaluation and follow-up

According to the National Cancer Institute Common Terminology Criteria for Adverse Events (NCI CTCAE) v5.0 criteria (National Institutes of Health National Cancer Institute, 2017), the scoring was performed from the beginning of conditioning to the myeloid reconstitution. The toxic side effects of the conditioning regimen were observed, including nonhematological toxicity reactions, such as gastrointestinal reaction, nephrogenic hepatic dysfunction, hemorrhagic cystitis, hepatic veno-occlusive disease, neurotoxicity, and infection; hematological toxicity, duration of neutrophil deficiency, assessment of hematopoietic recovery time.

After auto-HSCT, treatment evaluation was proformed serially for each patient by bone marrow morphology, FQ-PCR, and multicolor flow cytometry at 2 weeks, 4 weeks, 6 weeks, 2 months, 3 months, 6 months, 9 months, 12 months, 15 months, 18 months, 21 months, 24 months, 30 months, 36 months, 48 months and 60 months. All patients were followed up by outpatient and inpatient medical records and telephone. Statistical data included OS, leukemia-free survival (LFS), relapse, transplant-related death, hematopoietic reconstitution, etc. OS time: the time from hematopoietic stem cell infusion date to death or the last follow-up; LFS time: the time from hematopoietic stem cell infusion date to relapse, death or the last follow-up; relapse time: the time from hematopoietic stem cell infusion date to MRD positive and/or the emergence of extramedullary leukemia; transplant-related deaths: other deaths not related to relapse.

### Statistical analysis

All procedures performed in studies involving human partipants were in accordance with the ethical 80 standards of the institutional and national research committee (Ethics committee of Blood Disease Hospital, Chinese Academy of Medical Sciences). The institutional review board approved all study procedures and forms. SPSS 26.0 was used for statistical analysis. OS rate, relapse rate, and LFS rate was estimated using the Kaplan-Meier method and compared between arms using the log-rank test. *p*-value less than 0.05 was considered statistically significant.

## Results

### Patient characteristics

A total of 22 AML patients were enrolled (Table 1). Two AML-M3 patients were at CR2, and the remaining 20 patients were at CR1, including 12 patients who achieved negative MRD in 1 course of chemotherapy, 5 patients who achieved negative MRD in 2 courses of chemotherapy, 1 patient who achieved negative MRD in 3 courses of chemotherapy, and 2 patients who achieved negative MRD in 4 courses of chemotherapy. All patients had negative MRD before transplantation.

**TABLE 1:** Clinical characteristics of 22 patients.

Case data	Number of patients (%)
Median age (years)	32(range 15–54)
Gender	
Male	13(59.09%)
Female	9(40.91%)
AML (non-APL)-CR1	20(90.91%)
Risk group#: favorable group	10(45.45%)
intermediate group	10(45.45%)
AML-M3-CR2	2(9.09%)
Karyotype at onset	
Normal	14(63.64%)
Abnormal	6(27.27%)
No mitotic phase seen	1(4.55%)
Unknown	1(4.55%)
Median course of chemotherapy before transplantation	4 (range 2–6)*
The number of chemotherapy courses achieved MRD negative	
1 courses	12(54.55%)*
2 courses	5(22.73%)*
3 courses	1(4.55%)*
4 courses	2(9.09%)*
Median time from diagnosis to stem cell transplantation (months)	7(range 5–10)*
conditioning regimen	
BU + CY + CLAD+ Ara-c	14(63.64%)
BU + CLAD+ Ara-c	6(27.27%)
BU + Mel + CLAD+ Ara-c	2(9.09%)
Median number of reinfused mononuclear cells (×10^8^/kg)	10.00(range 2.88–20.97)
Median number of reinfused CD34^+^ cells (×10^6^/kg)	1.89(range 1.52–10.44)
Median time of granulocyte implantation (days)	13(range 10–34)
Median time of platelet implantation (days)	28(range 14–113)

^#^According to ELN 2017 classification (See Table 2 for details).

^*^Excluding two cases of M3.

**TABLE 2:** Risk group of 20 patients.

Patient	Diagnosis	Cytogenetics	Molecular genetics	According to ELN 2017 classification
1	AML-M2a	unknown	Wild-type NPM1 without FLT3-ITD	Intermediate
2	AML-M2a	normal	CBFB-MYH11	Favorable
3	AML-M5	normal	Wild-type NPM1 without FLT3-ITD	Intermediate
4	AML-M2a	normal	Wild-type NPM1 without FLT3-ITD	Intermediate
5	AML-M1	normal	Wild-type NPM1 without FLT3-ITD	Intermediate
6	AML-M5b	normal	Wild-type NPM1 without FLT3-ITD	Intermediate
7	AML-M2a	normal	Wild-type NPM1 without FLT3-ITD	Intermediate
8	AML-M5b	normal	Mutated NPM1 without FLT3-ITD	Favorable
9	AML-M2a	normal	Wild-type NPM1 without FLT3-ITD	Intermediate
10	AML-M4	normal	Mutated NPM1 without FLT3-ITD	Favorable
11	AML-M2b	45,X,-Y,t(8; 12; 21)(q22; q24; q22)(20)	RUNX1-RUNX1T1	Favorable
12	AML-M2a	normal	Wild-type NPM1 without FLT3-ITD	Intermediate
13	AML-M5a	normal	Mutated NPM1 and FLT3-ITD^high^	Intermediate
14	AML-M5	normal	Wild-type NPM1 without FLT3-ITD	Intermediate
15	AML-M2a	normal	Biallelic mutated CEBPA	Favorable
16	AML-M2b	45,X,-Y,t(8; 21)(q22; q22)(12)/46,XY(8)	RUNX1-RUNX1T1	Favorable
17	AML-M5	no mitotic phase seen	Mutated NPM1 without FLT3-ITD	Favorable
18	AML-M2b	45,X,-Y,add(7)(p22),der(8)add(8)(?p23) t(8; 21)(q22; q22)(20)	RUNX1-RUNX1T1	Favorable
19	AML-M5	normal	Mutated NPM1 without FLT3-ITD	Favorable
20	AML-M4eo	46,XX,inv(16)(p13; p22)(6)/46,XX(14)	CBFB-MYH11	Favorable

### Conditioning-related toxicity and transplantation-related complications

During conditioning, all the 22 patients had gastrointestinal adverse reactions without exceeding grade 3 (Table 3), and 6 patients had liver damage less than grade 2, which were alleviated after liver and gastric protection treatment. Before myeloid reconstitution, 4 cases of bloodstream infection, 2 cases of pulmonary infection, 4 cases of intestinal infection, 7 cases of perianal infection, 5 cases of oral infection, and 2 cases of paronychia were alleviated after active anti-infection treatment. No hemorrhagic cystitis, hepatic veno-occlusive disease, or neurotoxicity occurred; there were no renal dysfunction or transplantation-related deaths.

**TABLE 3:** Summary of adverse events.

Adverse event	Number of patients (%)				
Grade of adverse events	grade 1	grade 2	grade 3	grade 4	grade 5
Hematologic					
Thrombocytopenia	0	0	0	22 (100.00%)	0
Leukopenia	0	0	0	22 (100.00%)	0
neutropenia	0	0	0	22 (100.00%)	0
Anemia	0	0	22 (100.00%)	0	0
Nonhematologic					
Febrile neutropenia	0	0	22 (100.00%)	0	0
Infection	0	0	22 (100.00%)	0	0
Diarrhea	5 (22.73%)	2 (9.09%)	3 (13.64%)	0	0
Nausea	0	22 (100.00%)	0	0	0
Mucositis or stomatitis	0	5 (22.73%)	0	0	0
Increased aminotransferase	4 (18.18%)	2 (9.09%)	0	0	0
Pneumonitis or pulmonary infiltrates	0	2 (9.09%)	0	0	0

### The number of autologous stem cell infusions and hematopoietic reconstitution

The median number of returned mononuclear cells was 10.00 (2.88–20.97)×10^8^/kg, and the median number of CD34^+^ cells was 1.89 (1.52–10.44)×10^6^/kg. Hematopoietic reconstitution was achieved in all patients, with a median time of 13 (10–34) days for neutrophil reconstitution and 28 (14–113) days for platelet reconstitution.

### Survival analysis

**FIGURE 1:**
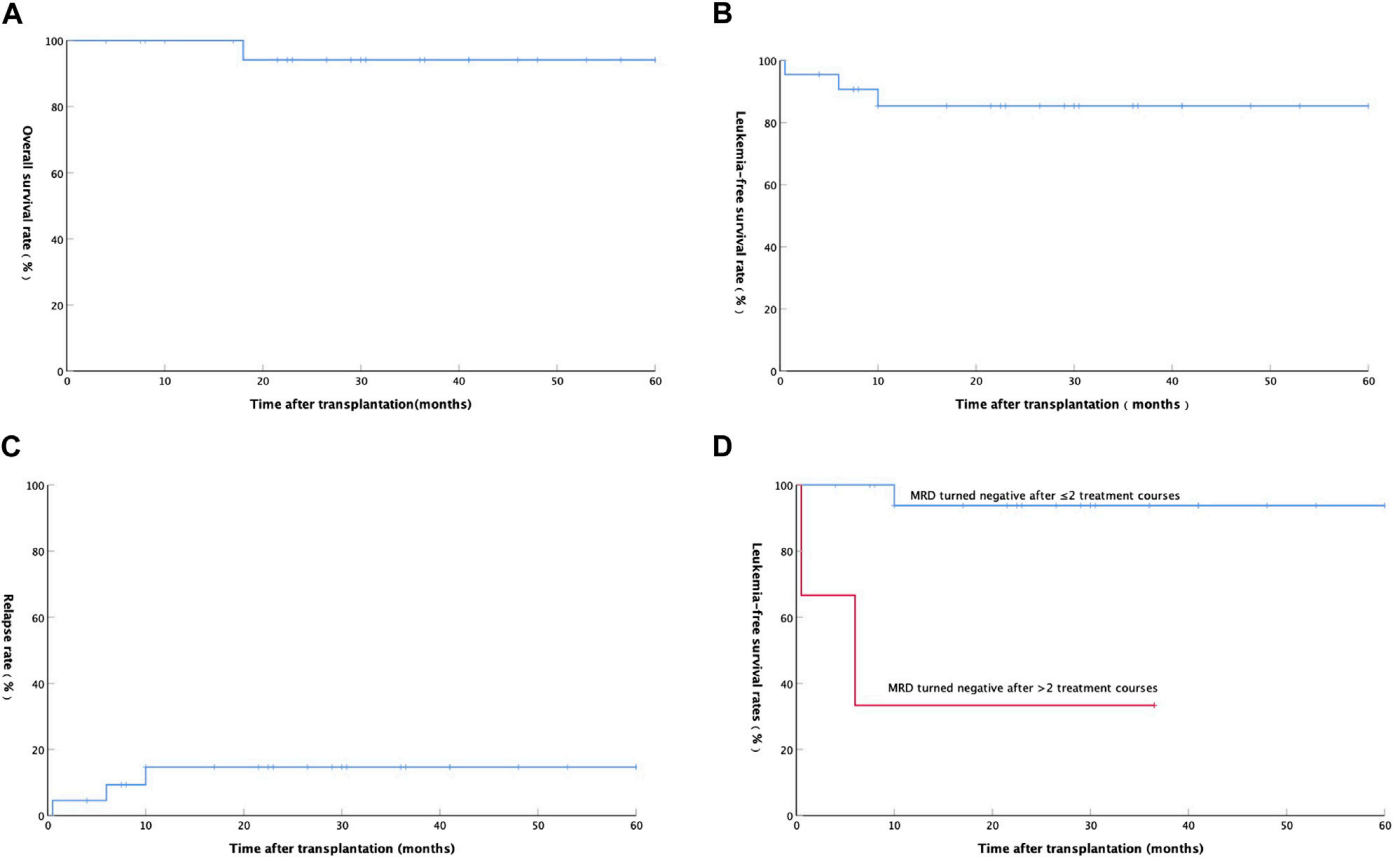
**(A)** Overall survival rate of 22 AML patients after auto-HSCT; **(B)** Leukemia-free survival rate of 22 AML patients after auto-HSCT; **(C)** Relapse rate of 22 AML patients after auto-HSCT; **(D)** Relationship between Leukemia-free survival rate and minimal residual disease in 20 patients with AML(except APL).

At a median follow-up time of 29.5 (ranging from 4 to 60) months until mid-September 2021, 21 of the 22 patients were alive, and the remaining 1 patient died due to relapse. The estimated 2-year OS, LFS, and relapse were 94.1 ± 5.7% (Figure 1A), 85.3 ± 7.9% (Figure 1B), and 14.7 ± 7.9% (Figure 1C), respectively.

A total of 3 (13.64%) patients relapsed after transplantation at a median time of 6 (ranging from 0.5 to 10) months. The first patient with hematologic relapse after auto-HSCT received an allo-HSCT and achieved complete hematologic remission and full donor chimerism with prolonged survival. The second patient with hematologic relapse after auto-HSCT was treated with a FLAG [fludarabine (FLU) 30 mg/m^2^/d, d1-5; Ara-c 1 g/m^2^/d, d1-5; G-CSF 200 ug/m^2^/d, d0-5] chemotherapy. Unfortunately, the patient did not achieve remission and received transfusion of blood products, anti-infection and other supportive treatments, and then died at 18 months after transplantation. The third patient relapsed with positive MRD was treated with interleukin-2 (100 wu/d, iH QOD) and interferon (300 wu/d, iH QOD) alternately and then he achieved and maintained MRD negative. Among the 20 AML patients without APL, LFS was significantly better in patients with negative MRD after 1 or 2 courses than patients with negative MRD after more than 2 courses (*p* = 0.001, Figure 1D).

## Discussion

Auto-HSCT for AML in favorable- and intermediate-prognosis groups recieced more attention due to improvement of chemotherapy efficacy and detection level of residual diseases. In Vellenga’s study (Vellenga et al., 2011) 259 AML patients at CR1 received combination chemotherapy and other 258 patients received auto-HSCT consolidation; the 5-year relapse rate in the auto-HSCT group was significantly lower than that in the combination chemotherapy group (58% vs. 70%, *p* = 0.02); the 5-year LFS rate was better in the auto-HSCT group (38% vs. 29%, *p* = 0.065). In Mizutani’s study (Mizutani et al., 2016), the 5-year OS rate of AML patients with CR1 in the auto-HSCT group(n = 375) was significantly better than that in the allo-PBSCT group(n = 380) (*p* = 0.004). Other studies reported similar results (Usuki et al., 2012; Keating et al., 2013; Yao et al., 2017). The encouraging results were due to the low transplantation-related mortality, low infection complications, and a high quality of life for patients after auto-HSCT.

It should be noted that auto-HSCT has an increased risk of relapse after transplantation due to its lack of graft-versus-leukemia effect. Reducing relapse after transplantation is the key to improving the efficacy of autologous transplantation.

CLAD is a purine nucleoside analog that can kill both proliferating and nonproliferating cells; it can exert demethylation by indirectly inhibiting DNA methylation transferase and depleting the methyl donor, thus promoting cells apoptosis (Vahdat et al., 1994; Robak and Wierzbowska, 2014; Freyer et al., 2015). Additionally, the combination of CLAD and Ara-C can increase the uptake of Ara-C and increase the intracellular content of Ara-C, which enhances the cytotoxicity and antitumor effect of Ara-C (Ramos et al., 2015). Common adverse responses of CLAD are myelosuppression, immunosuppression, and infection. After treatment, oral and perianal care should be strengthened to avoid infection. In Holowiecki’s study (Holowiecki et al., 2012) a total of 652 newly diagnosed adult AML patients were prospectively randomized into three groups and were treated with standard DA, standard DA + CLAD, and standard DA + FLU for induction therapy. After remission, the treatment regimen was the same in the three groups, with a median follow-up of 2.8 years. In this study, the results confirmed that CR and OS rates were significantly improved in the CLAD group. Bao’s study (Bao et al., 2018) showed the CR rate (61.7% vs. 48.7%) and 3-year OS rate (35.3% vs. 26.8%) were better in the CLAG (CLAD+ Ara-C +G-CSF) chemotherapy group than those in the FLAG(FLU + Ara-C + G-CSF) group for 103 patients with refractory relapsed AML.

Based on the above study, for 22 AML patients with negative MRD without suitable donors, we applied an improved conditioning regimen of CLAD and Ara-C to perform auto-HSCT as a consolidation treatment for patients after CR. Nonhematological toxicity during conditioning was mainly gastrointestinal reactions, hepatic injury, and infection, all of which were without exceeding grade 3 and were alleviated after active treatment. In terms of hematologic toxicity, all patients had grade IV myelosuppression after conditioning to achieve the purpose of myeloid removal, and all patients obtained hematopoietic reconstitution after transplantation with a median time to neutrophil and platelet reconstitution of +13 d and +28 d respectively, which was consistent with literature reports (Simancikova et al., 2017; Helbig et al., 2018). No transplantation-related deaths were recorded. One patient who relapsed after auto-HSCT and did not achieve remission with combination chemotherapy died at the 18th months after transplantation. The other 21 patients survived at the end of follow-up. Nineteen of the surviving patients had negative MRD after auto-HSCT, and the other 2 patients achieved negative MRD after maintenance chemotherapy or allo-HSCT.

Our study also demonstrated that AML patients with negative MRD after 1-2 courses of chemotherapy had better outcomes after auto-HSCT; the estimated 2-year OS, relapse, and LFS rates were 94.1 ± 5.7%, 14.7 ± 7.9%, and 85.3 ± 7.9%, respectively. In Yao’s study (Yao et al., 2017), 46 adult AML patients at CR1 underwent auto-HSCT with the conditioning regimen Bu + Cy + Flu + Ara-c or Bu + Flu + Ara-c; the 3-year OS, LFS and relapse rates were 84.4%, 85.4% and 5.0% in the favorable-risk group, and 73.7%, 69.1% and 30.9% in the intermediate-risk group, respectively. In comparison, the overall efficacy of our study on auto-HSCT with CLAD-combination conditioning regimen was better than FLU-combination conditioning regimen; the overall efficacy of our study was also better than that reported in the previous literature (Saraceni et al., 2017; Simancikova et al., 2017; Helbig et al., 2018).

The higher overall efficacy of the patients in our study may be related to the following factors: (1) all patients in the study were at CR1 except 2 AML-M3 patients at CR2, and all patients had negative MRD before transplantation, and 17 AML patients with non-APL had negative MRD after 1 or 2 course of consolidation therapy; Both MRD status before auto-HSCT and MRD status after 1 course of consolidation therapy were independent prognostic factors affecting OS and LFS after auto-HSCT (Mulé et al., 2016; Yao et al., 2017; Buccisano et al., 2019; Percival and Estey, 2019); (2) compared with FLU, the addition of CLAD to the conditioning regimen enhanced the anti-leukemia effect of conditioning; (3) maintenance therapy with IL-2, interferon or azacytidine was performed for most patients with a intermediate prognosis, which may reduce the relapse after transplantation and improve the overall efficacy. Of course, our single-center study also has some shortcomings, such as a small number of cases, a nonrandomized controlled study, young age of patients, use of maintenance therapy in 8 patients post-transplantation, addition of APL patients to cohort and a short follow-up period.

In conclusion, for AML patients with negative MRD and no suitable donor, a CLAD-combination conditioning regimen might enhanced the anti-leukemia effect of conditioning without increasing conditioning-related toxicity. The rate of OS and LFS after transplantation was high, which can be used as effective conditioning for auto-HSCT in AML patients. In addition, AML patients with a favorable prognosis and intermediate prognosis at CR1, especially those with negative MRD after one course of consolidation therapy, showed better efficacy after auto-HSCT.

## Data Availability

The original contributions presented in the study are included in the article/supplementary materials, further inquiries can be directed to the corresponding authors.
